# Microhydration and the Enhanced Acidity of Free Radicals

**DOI:** 10.3390/molecules23020423

**Published:** 2018-02-14

**Authors:** John C. Walton

**Affiliations:** EaStCHEM School of Chemistry, University of St. Andrews, St. Andrews KY16 9ST, UK; jcw@st-andrews.ac.uk; Tel.: +44-(0)1334-463864

**Keywords:** free radicals, acidity, DFT computations, hydration

## Abstract

Recent theoretical research employing a continuum solvent model predicted that radical centers would enhance the acidity (RED-shift) of certain proton-donor molecules. Microhydration studies employing a DFT method are reported here with the aim of establishing the effect of the solvent micro-structure on the acidity of radicals with and without RED-shifts. Microhydration cluster structures were obtained for carboxyl, carboxy-ethynyl, carboxy-methyl, and hydroperoxyl radicals. The numbers of water molecules needed to induce spontaneous ionization were determined. The hydration clusters formed primarily round the CO_2_ units of the carboxylate-containing radicals. Only 4 or 5 water molecules were needed to induce ionization of carboxyl and carboxy-ethynyl radicals, thus corroborating their large RED-shifts.

## 1. Introduction

A transient-free radical is necessarily reactive at the site (X) of its unpaired electron (upe). In addition, a free radical with a structure containing a proton donor group may undergo ionic dissociation to a radical anion and a proton:

^•^XH_2_WAH→^•^XH_2_WA^−^ + H^+^ in which W is a connector or spacer group, and A is the site of the departing proton. Effectively, the free radicals in this category can function as Brφnsted acids. More than 40 years ago, Hayon and Simic drew attention to the fact that free radicals could be more acidic than parent compounds [1]. Other pulse radiologists, particularly Steenken and co-workers, demonstrated analogous behavior for the radical cations of phenols and nucleosides [2,3]. Recently, several situations have been recognized in which the radical enhancement of proton dissociations has been of key importance. For example, Buckel, Zipse, and co-workers identified facile deprotonation of a β-keto-alkyl radical as the key step in an enzyme catalyzed dehydration of (*R*)-2-hydroxyglutaryl-CoA [4,5]. A similar key step was proposed in the analogous enzyme-catalyzed dehydration of (*R*)-2-hydroxy-4-methylpentanoyl-CoA [6]. Cyclohexadienyl type radicals are intermediates in numerous base-promoted homolytic aromatic substitution (BHAS) reactions; Studer and Curran have pinpointed their enhanced acidity as the driving force for these processes [7]. In addition, Radom and co-workers have published theoretical studies showing that, in general, CH_2_WOH radicals are more acidic than their parent CH_3_WOH alcohols [8,9].

A recent theoretical investigation of radicals containing carboxylic acid groups gave guidance as to what structural features are required for proton loss to be enhanced. The size of the effect and the identity of the connector groups that do and do not transmit the enhancement were also studied [10]. The term “RED-shift” was adopted as a short and convenient acronym for the phenomenon of Radical Enhancement of Dissociation. Appreciable RED-shifts were shown to occur for radicals dissociating to conjugate radical anions that allowed the displacement of an electronic charge away from their formal anionic centers coupled with displacement of spin density away from their formal radical centers.

A convenient measure of the RED-shift was found to be:

Δp*K*_a_ = p*K*_a_(model) − p*K*_a_(radical) (1)

in which the models [HXH_2_WAH] were structurally identical to the radicals [^•^XH_2_WAH], except that the unpaired electrons had been replaced by -*H*-atoms.

The transient nature of most free radicals makes experimental determination of their p*K*_a_ values difficult. Consequently, comparatively few radical acid dissociation constants have been measured, and error limits are necessarily high. Fortunately, computational methods for estimating p*K*_a_s have been developed, and some of these refer directly to carboxylic acids [11,12,13,14,15,16]. These QM methods usually rely on computation of the free energies of deprotonation ΔG_A-HA_ and, with this intent, a DFT functional suitable for C-centered free radicals was developed [17]. This method enabled several radicals that had large Δp*K*_a_ values to be pinpointed [10,18]. In this DFT method, the solvent (water) was allowed for simply by means of the CPCM continuum model [19]. A real possibility is that continuum solvent models would not be reliable for small and strong acids. Specific interactions of the solvent with the conjugate radical anions seem particularly likely because of their high charge densities. It is known that strong mineral acids spontaneously ionize in association with just a few microsolvating water molecules. For instance, both experiment and DFT computations showed that HCl dissociated into Cl^−^(H_2_O)_3_(H_3_O^+^) upon association with just four water molecules [20], and similar behavior was observed for other strong acids [21]. In fact, it has been noted that as the acidity of an acid increases, so the number of water molecules required to induce ionization (*N_G_^i^*) tends to decrease [17,22].

**Scheme 1:**
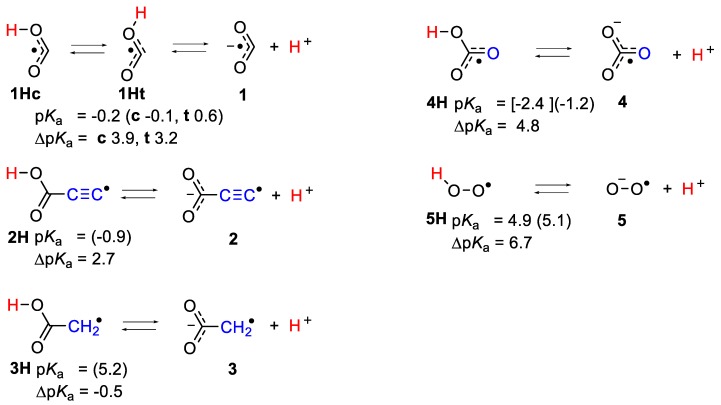
Set of Acid Radicals for Microhydration Study. Experimental p*K*_a_ values with DFT computed values in parenthesis.

Theoretical studies of the microhydration of acids are challenging because the number of possible 3D arrays increases steeply as the number of water molecules increases. The potential energy surfaces (PES) rapidly become complex with many shallow minima. Distinguishing the global minimum from nearby local minima becomes problematic. Microhydration studies of transient acid radicals have been published for the bicarbonate radical **4H** [17] and the hydroperoxyl radical **5H** [23] (see Scheme 1). Some recent studies have shown that the limitations of the continuum models can be overcome with microhydration. For example, Close and Wardman studied di-hydration of the tyrosyl radical and discussed related structures [24]. The objectives of the present computational study were to investigate the effect microhydration has on the enhancement of acidity observed for specific free radicals with and without RED-shifts. Are the increased acidities computed with the continuum solvent model real? What effect does the upe in the radical have on the solvent microstructure? Can the number of water molecules needed to induce ionic dissociation (*N_G_^i^*) be determined for RED-shifted radicals?

## 2. Results and Discussion

A set of acid radicals with particular structural features was chosen for microhydration study and is shown in Scheme 1, together with experimental and computed p*K*_a_ and Δp*K*_a_ values [10]. Three of the set contain carboxylic acid functional groups with differing spacer units. The carboxyl radicals **1Hc** & **1Ht** are very strong acids with large RED-shifts (Δp*K*_a_ = 3.9 & 3.2, respectively), and contain the upe *formally* right on the carboxylate *C*-atom. In carboxy-ethynyl radical anion **2,** the upe and negative charge are *formally* separated by an ethynyl unit, which proved to be a very efficient conductor of RED-shift. In carboxy-methyl radical anion **3,** the upe and negative charge are *formally* separated by a methylene unit that completely negated RED-shift. The set also contains the bicarbonate radical anion **4** that has the upe and carboxylic acid group *formally* separated by an *O*-atom. The final member, hydroperoxyl **5H**, has a unique structure such that the upe and charge are *formally* on adjacent *O*-atoms. The ease of deprotonation of the acid radicals will be strongly influenced by solvation, which will stabilize the conjugate radical anions produced during dissociation more than the neutral radical precursors.

A benchmarking study involved comparison of 12 radical reaction types and 23 different DFT functionals (plus the MP2 ab-initio method), with the high level composite ab initio G4 method [25]. The CAM-B3LYP functional incorporating the Coulomb-attenuating method [26] gave lowest mean absolute deviations (MAD) and performed best with radical species [17]. Microhydration of the bicarbonate radical **4H,** which has a large RED-shift, was investigated using this approach; cluster configurations were obtained for [HOC(O)O^•^·*n*H_2_O] with *n* = 1 to 8. That **4H** was a very strong acid was confirmed by the finding that partial ionization spontaneously occurred with only 4 water molecules, and ionization was complete for 5 microsolvating water molecules, i.e., *N_G_^i^* = 5. The p*K*_a_ of **4H** obtained with this computational method (−1.2) was in satisfactory agreement with other estimates (see Scheme 1 and reference [17]). For each of the other radicals in Scheme 1 (^•^XH_2_WAH), optimized structures were obtained for hydrated clusters (^•^XH_2_WAH.*n*H_2_O) where *n* was increased until spontaneous dissociation (^•^XH_2_WA^−^.*n*H_2_O.H^+^) took place for some value of *n* (=*N_G_^i^*). Microhydrated cluster structures published by Maity and co-workers for structurally analogous formic [27] and trifluoroacetic acid [28] and by Leopold for various acids [21] served as useful models for the starting points for optimizations. The larger hydration clusters (*n* > ~8) displayed potential energy surfaces with quite a lot of shallow local minima, such that extensive searching was needed to find the global minima.

### 2.1. Microhydration of Carboxyl Radicals ***1Hc*** and ***1Ht***

Carboxyl radicals are important in combustion processes and in the oxidation cycle of Earth’s atmosphere because of their formation from the reaction of CO with hydroxyl radicals. The p*K*_a_ of **1H** was determined to be −0.2 in a pioneering EPR spectroscopic study by Fessenden and co-workers [29]. The radical’s structures and reactions have also been studied experimentally by vibrational [30,31,32] and rotational spectroscopy [33,34,35], as well as by a number of high level QM computational methods [36,37]. Experiment and theory indicate that they exist as a pair of *cis*- and *trans*-conformers **1Hc** and **1Ht** separated by a torsional barrier of about 7.7 kcal/mol and with **1Ht** about 1.7 kcal/mol lower in energy [35].

**Figure 1:**
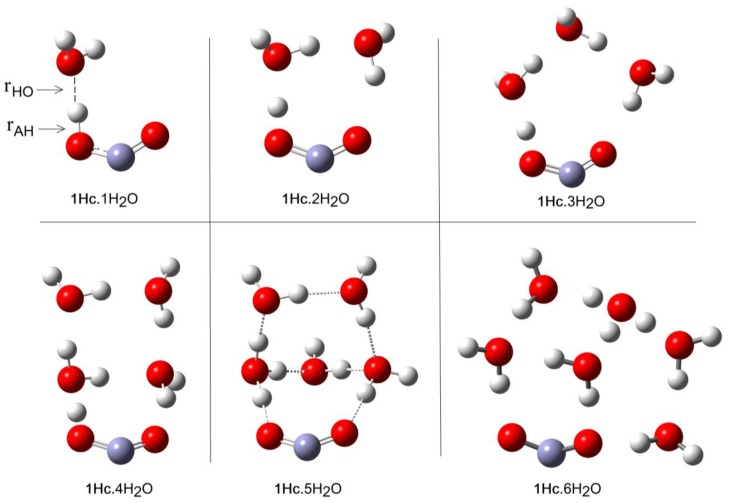
Optimized Cluster Structures of *cis*-Carboxyl (**1Hc**) with 1 to 6 H_2_O molecules.

Microhydration of the *cis*-conformer [**1Hc**.nH_2_O] was examined for *n* = 1 to 8 and of the *trans*-conformer **1Ht** for *n* = 1 to 12. The DFT method with the CAM-B3LYP functional and the 6-311+G(2d,p) basis set (see above) and including the CPCM solvent continuum model was employed. The PES for each value of *n* showed a series of minima and the global minimum energy cluster structures were obtained. Figure 1 shows the global minimum energy cluster structures for the *cis*-conformer associated with 1 to 6 H_2_O molecules.

**Figure 2:**
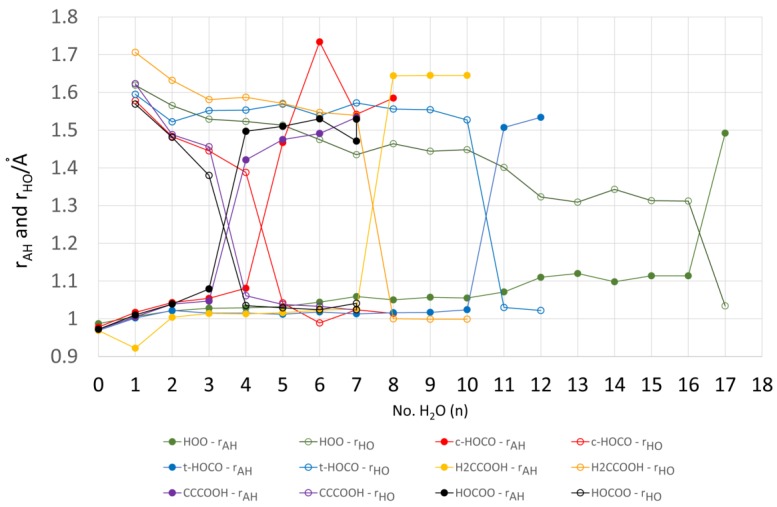
Microhydrated acid radicals (DFT optimized structures): plots of computed distances r_AH,_ and to the nearest water r_HO_, versus the number of solvating waters (*n*). Data for **4H** (HOCOO, black), **1Ht** (*t*-HOCO, blue), **1Hc** (*c*-HOCO, red), **3H** (H_2_CCOOH, yellow), **2H** (CCCOOH, purple), and **5H** (HOO, green).

For each value of *n* (except *n* = 1), a series of initial structures was tested. Two minima were found for 2 × H_2_O clusters, three minima were obtained for 3 × H_2_O clusters, and ten were obtained for the 4 × H_2_O clusters. In the latter case, several dissociated ion pairs were located as local minima [**1**.H^+^ 4H_2_O], and one of these was only 4.1 kcal/mol higher in energy than the global undissociated minimum shown in Figure 1. The minimum number of water molecules needed to obtain an ion pair as a local minimum was therefore *N_L_^i^* = 4*.* Ten minima were found for the 5 × H_2_O cluster and in this case the global minimum structure was an ion pair (Figure 1). Thus, the number of water molecules needed to render the hydrated ion pair [**1**. H^+^5H_2_O] a global minimum was *N_G_^i^* = 5. As expected, the number of possible local minima increased steadily with cluster size. For the structures of the global minimum clusters, the computed lengths of the radical acid A–H bonds (OC^•^O–H), r_AH_, and the lengths of the bonds from the leaving proton to the nearest H_2_O molecule, r_HO_, (see Figure 1) were useful indicators. These distances are plotted as a function of the number of H_2_O molecules in the global minimum clusters for radical **1Hc** in Figure 2. The graph illustrates that r_AH_ remained close to 1.0 Å in the 1× to 4 × H_2_O clusters and increased steeply to 1.47 Å in the ionized 5 × H_2_O cluster. This behavior was practically mirrored by the distances from the nearest water to the leaving proton (r_HO_), which remained in the 1.6 to 1.4 Å range for the 1 × to 4 × H_2_O clusters before steeply decreasing to 1.04 Å in the 5 × H_2_O cluster (see Figure 2).

**Figure 3:**
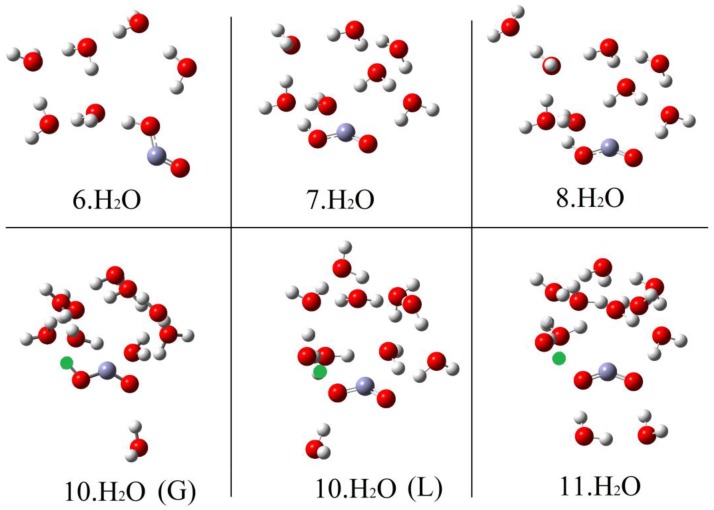
Optimized Cluster Structures of *trans*-Carboxyl (**1Ht**) with 6 to 11 H_2_O molecules. Departing proton shown in green.

Hydration of the *trans*-conformer **1Ht** took a rather different course. As with **1Hc,** a series of local minima was obtained for each hydration level. Cluster structures were more open, particularly for mono- to penta-hydration. A significantly higher level of hydration was required to induce ionization. Global energy minimum structures are illustrated in Figure 3 for selected clusters of **1Ht** with 6 to 11 H_2_O molecules.

No clusters containing ionized **1Ht** were obtained for 1× up to 9 × H_2_O components. For these clusters, the radical to proton bond length r_AH_ remained steady at about 1.02 Å and the proton to water distance r_HO_ also remained around 1.5 to 1.6 Å (see Figure 2). For 10 microsolvating H_2_O molecules, 9 minima were obtained including a local minimum only 2.3 kcal/mol above the global minimum, in which **1Ht** was ionized (see Figure 3 for local and global minima). Finally, with 11 microsolvating H_2_O molecules, the global minimum structure contained the fully ionized radical [**1**.11H_2_O,H^+^] (Figure 3). Thus, for the *trans*-carboxyl radical *N_L_^i^* = 10 and *N_G_^i^* = 11.

At first, the much larger *N_G_^i^* for *trans* **1Ht** than for *cis* **1Hc** seemed surprising. In the case of **1Hc,** the *cis* arrangement of the HO group with the adjacent C–O bond was a crucial feature. This enabled a first water molecule to *H*-bond to this OH and a second water molecule to simultaneously *H*-bond to the first water and to the carboxyl C–O, thus forming a 5-*O*-atom ring (see Figure 1, **1Hc**.2H_2_O). Additional waters could then add whilst keeping ring structures intact. Finally, at *N_G_^i^* = 5, the structure evolved to an ionized 3D cage of rings in which the carboxyl moiety sensed two *H*-bonds and each water sensed at least two (**1Hc**.5H_2_O, Figure 1). This arrangement lowered the energy in relation to unionized clusters. With isomer **1Ht**, however, the *trans* arrangement of the H–O group with the adjacent C–O precluded the possibility of two waters simultaneously *H*-bonding to the carboxyl and to each other. A larger, more open ring of waters was needed to stretch from one side of the carboxyl group to the other (see **1Ht**.7H_2_O, Figure 3). In fact, the weaker *H*-bonds that resulted meant that four water *H*-bonds to the carboxyl were required before ionization could take place (see **1Ht**.11H_2_O, Figure 3).

**Figure 4:**
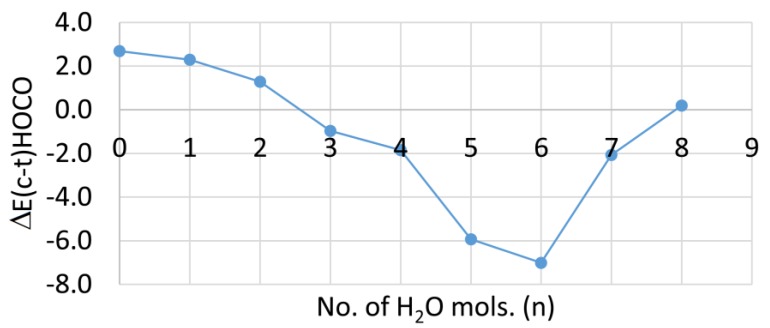
Graph of the Difference in the Energies (kcal/mol) of the **1Hc** and **1Ht** hydrated clusters as a function of the number of cluster water molecules.

As mentioned above, **1Ht** is lower in energy than **1Hc** in the gas phase. For the solvated clusters, the difference in DFT computed energies between **1Hc** and **1Ht** [ΔE(c–t)/kcal/mol] are plotted against the number of cage water molecules in Figure 4.

It is evident that the *trans**-***clusters are lower in energy for 0 to 2 cluster H_2_O, but that the *cis*-clusters are lower in energy for 3 to 7 microsolvating H_2_O molecules. In aqueous solution, therefore, thermodynamic control should ensure complete deprotonation by five waters. Hence, the experimental p*K*_a_ of −0.2 [29] probably corresponds to **1Hc**.

### 2.2. Microhydration of Carboxy-ethynyl ***2H*** and Carboxy-methyl ***3H*** Radicals

**Figure 5:**
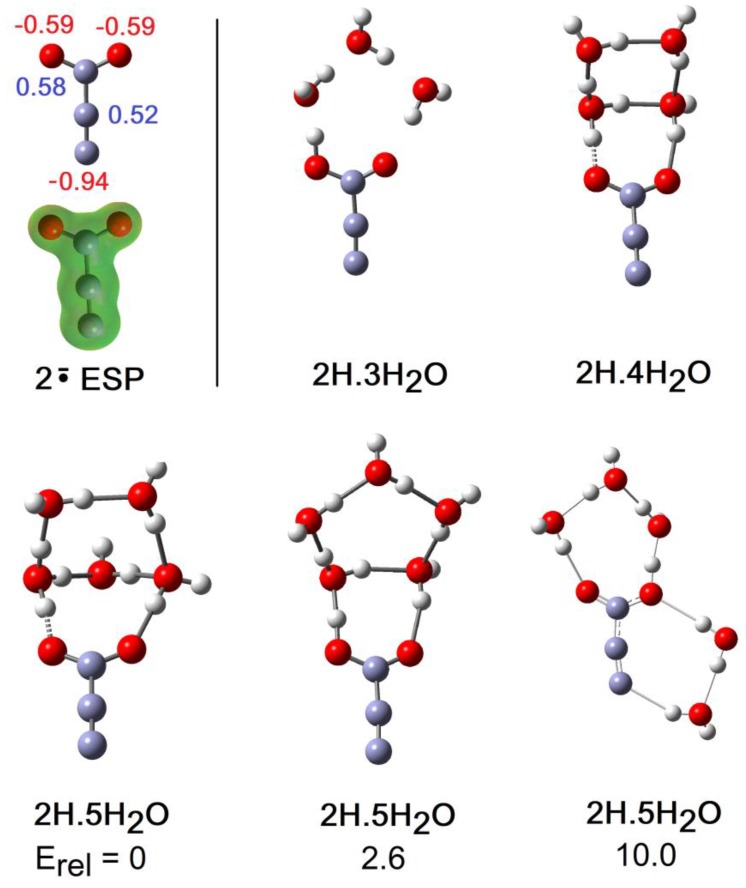
Optimized Structures of Hydration Clusters for Carboxy-ethynyl Radical **2H** and Radical Anion **2**. Mulliken charge: negative in red, positive in blue.

The formal structure of the carboxy-ethynyl radical anion **2** (Scheme 1) suggests the upe and charge are separated by the ethyne unit. However, DFT computations predicted **2H** to be a much stronger acid than propiolic acid (HC≡CCO_2_H, p*K*_a_ = 1.74) and hence to have a sizeable RED-shift [10]. Furthermore, much site exchange of spin and charge was computed for **2** with significant negative charge associated with the terminal ethyne *C*-atom. This was revealed by the Mulliken electronic charges (Figure 5), with the red numbers denoting negative charge. It followed that the normally hydrophobic C≡C unit might, in radical **2H**, take part in *H*-bonding to H_2_O, although the electrostatic potential surface (ESP, Figure 5) was fairly featureless, except for the negatively charged (red) *O*-atoms. It was of special interest therefore to examine microhydration of **2H** to check the prospect of the H_2_O clusters extending around to the C≡C terminus, as well as to confirm the enhanced acidity of this species. In the lowest energy structures for clusters with 3 and 4 H_2_O molecules (see Figure 5), *H*-bonding took place exclusively in the carboxyl unit. Addition of four H_2_O molecules caused spontaneous ionization, and the ionized structure was the global minimum. No ionized minima were found for clusters with only three H_2_O molecules and hence *N_L_^i^ = N_G_^i^* = 4. This was confirmed by the sudden increase in r_AH_ and decrease in r_HO_ for greater than 4 × H_2_O shown on Figure 2. The small *N_L_^i^* and* N_G_^i^* agree well with the high predicted acidity (negative p*K*_a_) for **2H**.

The global minimum structure on addition of 5 H_2_O molecules was the ionized cage structure shown in Figure 5. However, a unionized structure (Figure 5), obtained simply by expansion of the outer ring of the **2H**.4H_2_O cluster, was only 2.6 kcal/mol higher in energy. Another local minimum of the **2H**.5H_2_O system did exhibit *H*-bonding to the terminal atom of the ethyne unit as part of a ring connected to a carboxyl *O*-atom (Figure 5). However, this cluster was 10 kcal/mol higher in energy than the global minimum [38]. We deduce that *H*-bonding of H_2_O molecules to the ethyne unit of **2H** did not stabilize the conjugate radical anion **2**.

**Figure 6:**
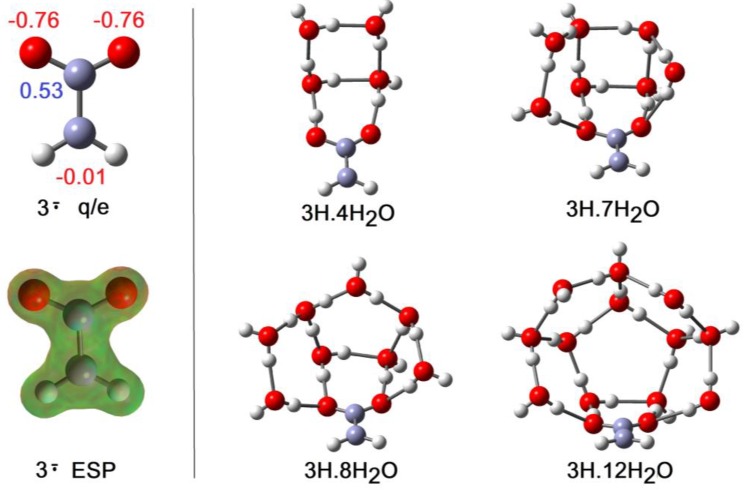
Optimized Structures of Hydration Clusters for Carboxy-methyl Radical **3H** and Radical Anion **3**. Mulliken charges: negative in red, positive in blue.

Although the charge and upe in the carboxy-methyl radical **3H** are *formally* separated by only the one *C*-atom of the methylene group, this radical was a much weaker acid than **2H** and comparable in strength to acetic acid. Unlike the ethyne unit, the CH_2_ spacer did not transmit any RED-shift to the carboxyl group. The computational results indicated negative electronic charges associated *only* with the carboxyl *O*-atoms and, in agreement, the ESP surface also showed no negative features adjoining the CH_2_ group (see Figure 6). In the cluster for **3H** with only a few H_2_O molecules, they *H*-bonded with the carboxyl group and the structures resembled the analogous clusters for **2H,** except that ionization was not observed. Compare, for example, **2H**.4H_2_O (ionized) of Figure 5 with **3H**.4H_2_O (undissociated) of Figure 6. In striking agreement with the lesser acidity and zero RED-shift of **3H**, ionized structures only appeared as local minima for **3H**.6H_2_O (3.6 kcal/mol above the global minimum) and **3H**.7H_2_O (1.6 kcal/mol above the global minimum), finally becoming the global minimum for **3H**.8H_2_O (see Figure 6). Figure 2 shows the abrupt increase in r_AH_ and abrupt decrease in r_HO _above 7 × H_2_O, and hence it follows that *N_L_^i^ =* 6 and* N_G_^i^* = 8. Investigations showed large numbers of local minima, differing in energy by < ~6 kcal/mol, for the **3H**.9H_2_O, **3H**.10H_2_O, **3H**.11H_2_O, and **3H**.12H_2_O clusters, with some ionized and some undissociated. No clusters involving *H*-bonding to the CH_2_ group were found, even with these larger water shells. The elegant optimum structure found for the **3H**.12H_2_O cluster (Figure 6) consisted of a symmetrical cage of 5-member *O*-atom rings, atop a planar **3H**.2H_2_O unit, forming part of a *quasi-*dodecahedron. The PES could only be partially explored for **3H**.12H_2_O, so it is not known if this is a local or global minimum.

### 2.3. Microhydration of Hydroperoxyl Radicals ***5H***

The hydroperoxyl radical/superoxide radical anion conjugate pair (**5H/5**) has been intensively studied over many years. It plays important roles in lipid peroxidations and in atmospheric chemistry [39,40,41,42,43,44]. The hydroperoxyl radical differs from the other acid radicals of this study in that it contains two adjacent *O*-atoms rather than the carboxyl unit. It was anticipated, therefore, that the hydrated clusters would have significantly different features. Microhydration of the hydroperoxyl radical **5H** was previously investigated by Novoa and co-workers for *n* = 1–4 and *n* = 10 using the HF/6-31++G(d,p) level of theory [23]. The adsorption and acid dissociation process of **5H** on the surface of (H_2_O)_20_ and (H_2_O)_21_ clusters were also studied computationally [45]. Not unexpectedly, the CAM-B3LYP/6-311+G(2d,p) method employed here, except for *n* = 1 and 2, gave somewhat different minimum energy structures for hydrated clusters.

**Figure 7:**
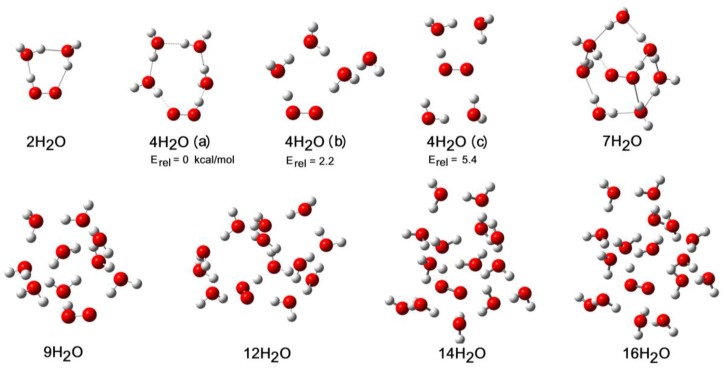
Optimum Structures of Selected HOO^•^ Radical/Water Clusters (**5H**.*n*H_2_O).

The minimum energy cluster structures of **5H**.*n*H_2_O were all un-ionized forms for *n* = 1 to 11; selected examples are in Figure 7 [the r_AH_ and r_HO_ lengths are in Figure 2]. The **5H**.2H_2_O structure was similar in essence to analogous carboxy 2×H_2_O clusters (above). A noteworthy feature for the 4xH_2_O clusters was that structure **5H**.4H_2_O(a) containing a 6-*O*-atom ring but only 5 *H*-bonds was lower in energy by 5.4 kcal/mol than structure (**c**) containing 6 *H*-bonds (Figure 7). The three *H*-bonds from waters to the OO unit in structure **5H**.4H_2_O(c) are all very long (1.874, 2.079, and 2.431 Å), probably because of angle strain in the two small 4-*O*-rings. In contrast, the *H*-bond from water to the OO unit in structure **5H**.4H_2_O(a) is shorter (1.853 Å) and the inter-water *H*-bonds (1.653, 1.712, and 1.737 Å) are considerably shorter than those of **5H**.4H_2_O(c). Structure **5H**.4H_2_O(b), which contains a 5-*O*-ring, is intermediate in energy, and the inter-water *H*-bonds (1.682, 1.786, 1.857 Å) are also intermediate in length.

3D cage structures were composed of rings of waters predominated for clusters with 7 or more waters and increased in complexity as more waters were added (see Figure 7). The 3D structures of the **5H**.14H_2_O and **5H**.16H_2_O clusters contained four H-bonds to the OO unit, and the number of inter-water *H*-bonds tended to a maximum.

**Figure 8:**
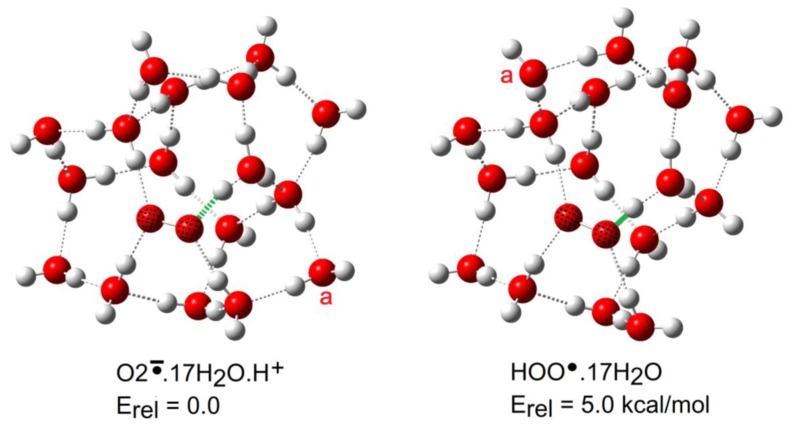
Ionized (left) and un-ionized (right) clusters of the hydroperoxyl radical (**5H**) with 17 H_2_O molecules. [Bond to departing H^+^ highlighted in green.].

The first cage containing ionized O_2_^−^^•^ and H^+^ appeared for *n* = 12, but was 4.3 kcal/mol higher in energy than the undissociated minimum energy structure. It was not possible to exhaustively explore the entire potential energy landscapes for *n* = 12, 13, 14, 15, and 16. For each of these clusters, both ionized and un-ionized forms were obtained, which differed in energy by < ~10 kcal/mol but with un-ionized cages as energy minima. For the **5H**.17H_2_O cluster, 11 local minima were obtained, with 9 being ionized and 2 un-ionized. The lowest energy ionized and un-ionized structures are illustrated in Figure 8. A remarkable result was the smallness of the structural difference that brought about spontaneous dissociation. The relocation of the single water molecule (labelled “a” in Figure 8) from one peripheral site to another was sufficient. This ostensibly minor change, together with concomitant bond length and angle reorganizations, caused a 5 kcal/mol lowering of the energy! *N_L_^i^* and* N_G_^i^* were estimated to be 13 and 17, respectively (see Figure 2). However, these larger hydration clusters probably existed as equilibria to which both ionized and un-ionized cages contributed. Under these circumstances, the “number of water molecules needed to induce ionization” (*N_G_^i^*) is probably a fuzzy concept and should probably not be considered as a sharply defined integer. In support of this, note the less abrupt change in the r_AH_ and r_HO_ distances for larger values of *n* in the **5H**.*n*H_2_O clusters as shown in Figure 2.

### 2.4. Microhydration, Acidity, and RED-Shift

**Figure 9:**
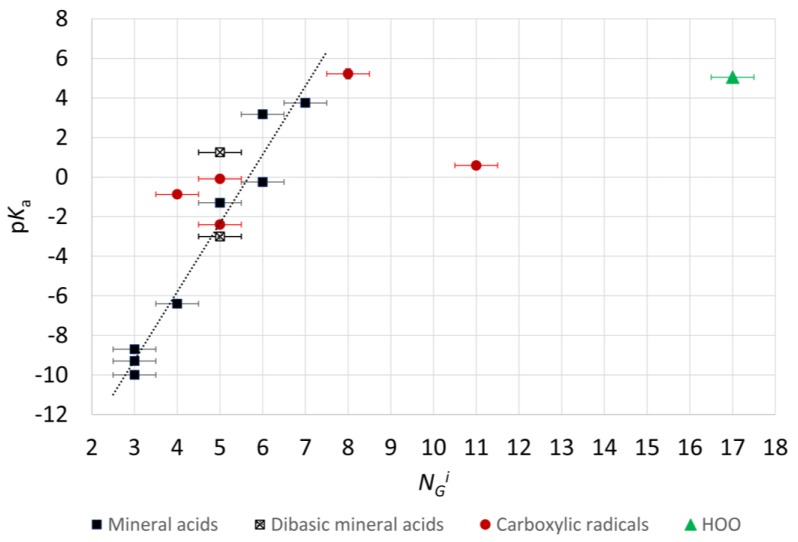
Plots of p*K*_a_ versus the number of microsolvating water molecules needed to cause ionization.

The computed numbers of water molecules needed to induce ionization in the radicals *N_G_^i^* are compared with literature data for mineral acids in Figure 9. Qualitatively, the data make good sense. Radicals **1Hc**, **2H,** and **4H** required only 4 or 5 water molecules to induce deprotonation, i.e., a similar number to acids such as HNO_3_ and CF_3_COOH. This agrees well with the previous computation of their p*K*_a_ values in the same range as mineral acids and gives an independent confirmation of the reality of their RED-shifts.

The *trans*-carboxyl radical **1Ht** was an obvious outlier and, as explained above, its greater *N_G_^i^* stems from the somewhat artificial *trans*-structure that, in a real solution, will convert to *cis*. The carboxy-methyl radical **3H** resembled a ‘normal’ carboxylic acid and needed the same number of water molecules to induce dissociation as formic (and probably acetic) acid. Thus, the microhydration study confirmed the lack of RED-shift for this radical. The data point for the HOO^•^ (**5H**) lies far away from those of the mineral and carboxylate-containing radicals. The radical character of **5H** greatly increases its acidity compared to that of H_2_O_2_. Structurally, however, the negative charge distributed to two adjacent *O*-atoms of superoxide is much more compact than on the two *O*-atoms of carboxylates. A large cage of water molecules was needed to stabilize this compact conjugate radical anion. That it diverges from the other acid radicals is not at all surprising.

## 3. Materials and Methods

DFT calculations were carried out using the Gaussian 09 suite of programs [46]. The CAM-B3LYP functional [26] with the 6-311+G(2d,p) basis set was employed for most species with the CPCM continuum model [19] with water as solvent. This model is derived from the COSMO model. Default values of the keywords Alpha, Radii, TSNUM, and TSARE were employed. Based on previous work with free radicals, the CAM-B3LYP functional, which combines the hybrid qualities of B3LYP with the long-range correction proposed by Tawada et al. [47], gave the best results in comparison with G4 (MAD: 2.5 kcal/mol. Functionals of comparable accuracy, such as BMK (MAD 3.2 kcal mol^−1^), ωB97 (MAD 3.3 kcal mol^−1^), and M05 (MAD 3.6 kcal mol^−1^), displayed no significant computational time advantages. Vibrational frequency calculations were implemented so that GS (no imaginary frequencies) and TS status could be checked (one imaginary frequency), and enthalpies and free energies were adjusted for zero point and thermal corrections to 1 atm and 298 K.

## 4. Conclusions

From this study of the structures of hydrated acid radicals, the following factors that reduced the energy of the hydrated species were identified: (i) *H*-bonding of water to the acidic proton of the radical; (ii) as many *H*-bonds from waters to the *O*-atoms of the radical as possible; (iii) as many *H*-bonds as possible (usually 2 or more) for each water molecule; (iv) rings of *O*-atoms (particularly 5- and 6-membered) were favored over chains or dangling waters for the larger hydrated clusters. The hydration clusters formed primarily round the CO_2_ units of the carboxylate-containing radicals and not round either of the CH_2_ or C≡C moieties that protruded from the cages. This held true even though the DFT study indicated considerable negative charge associated with the C≡C unit of the carboxy-ethynyl radical **2H**.

Although *trans*-carboxyl (**1Ht**) was lower in energy than **1Hc** in the gas phase, on microhydration the *cis*-clusters became lower in energy after only 3 solvating H_2_O molecules. In general, therefore, any carboxylic acid RCO_2_H in aqueous solution will probably convert to its *cis*-conformation and ionize from that.

It had previously been predicted that radicals **1Hc**, **2H,** and **4H** would be very strong acids with large RED-shifts [10,18]. The present finding, that just a few water molecules (4 to 5) were sufficient to induce spontaneous ionization of each one, was a valuable, independent corroboration of this. Interestingly, the RED-shift of the carboxy-ethynyl radical **2H** was found not to be due to solvation of its C≡C section. The increased stability of its conjugate radical anion **2** was due to other causes; probably stronger *H*-bonding to the carboxylate. Additionally, essentially zero RED-shift had been predicted for the carboxy-methyl radical **3H**, despite its rather similar structure. The microhydration study was again in full agreement and indicated that many more water molecules (8) were needed to induce ionization of **3H**.
